# Accuracy of Self‐Reported Cervical Screening Status Among Pregnant Women

**DOI:** 10.1111/ajo.70057

**Published:** 2025-11-26

**Authors:** Christine Thuy‐Trang Tran, Mandy Wang, Martin Plymoth, Judy Chen, Therese Mary McGee

**Affiliations:** ^1^ Department of Obstetrics and Gynaecology Westmead Hospital Sydney Australia; ^2^ Westmead Clinical School University of Sydney Sydney Australia; ^3^ Department of Medicine Westmead Hospital Sydney Australia; ^4^ Department of Clinical Microbiology Umeå University Umeå Sweden

**Keywords:** cervical screening, HPV screening, patient recall, pregnant women, self report

## Abstract

**Background:**

Pregnancy provides a special opportunity to improve cervical screening test (CST) uptake and reduce cervical cancer. Screening in Australia is free for Medicare‐eligible women ≥ 25 years if performed 5‐yearly, but not sooner. Either women's self‐reported last CST date or the National Cancer Screening Register (NCSR) can inform screening needs. However, accessing the NCSR is relatively difficult in public antenatal care.

**Aims:**

To assess if pregnant women's self‐reported last CST year is reliable in determining whether to offer CST in pregnancy or not.

**Methods:**

A retrospective Australian hospital study compared the self‐reported last CST recorded in the maternity database to NCSR records for all Medicare‐eligible women ≥ 25 years booked‐in for public antenatal care between 1 June and 30 November 2023.

**Results:**

The cohort (*n* = 1772) had median age 33 years (interquartile range 29–36). Nearly half (*n* = 862; 49%) were CST‐overdue/never‐screened. Self‐reported last CST dates were concordant with the NCSR for 80% (*n* = 1420) of participants in terms of needing (35%) or not needing (45%) a CST. However, 244 (14%) over‐reported being CST‐current when they were actually overdue/never‐screened, while 108 (6%) under‐reported their CST‐currency. Of the 862 women due for a CST, over‐reporting represented 28%. If clinicians relied solely on self‐reporting, these women would miss out on needed CST screening.

**Conclusion:**

Measures to improve the reliability of Australian women's self‐reported last CST are needed. This includes clinicians ensuring a woman always knows if a CST has been collected, the NCSR sending CST results to women (not just their practitioners) and promoting easier NCSR database accessibility for women.

## 1 Introduction

After the introduction of routine screening in 1991, Australia's rate of invasive cervical cancer fell 50% by 2003. Thereafter, it has plateaued [1]. In December 2017, primary screening in Australia transitioned from 2‐yearly Papanicolaou tests to a 5‐yearly human papilloma virus (HPV) cervical screening test (CST). The screening program includes an opt‐out National Cancer Screening Register (NCSR) linked to other government agencies. The NCSR sends screening invitations and reminders to Medicare‐eligible women aged 25–74 years [2]. Nationally, in 2023, about one quarter of the target group was not CST‐current [1]. Over 70% of Australia's nearly 1000 annual cervical cancer cases (peak age 35–45 years) occur in these unscreened and under‐screened women [1].

Medicare is Australia's universal health insurance scheme, available to citizens, permanent residents and limited others. Cervical screening for Medicare‐eligible women is free if performed at the recommended 5‐yearly interval. However, if carried out sooner than recommended (sooner than 4 years, 9 months), the woman (in private practice) or the hospital (public clinic) is billed around $60 Australian for the test (varies between labs).

The newly‐available option of self‐collect CST appears popular [3, 4] and is increasingly chosen by Australian women (40% of CSTs by the fourth quarter of 2024) [5]. Its availability makes screening in pregnancy more feasible, especially for midwives, leading to recent promotion of pregnancy as an ideal screening opportunity [6].

If clinicians are to appropriately target screening in pregnancy—while avoiding a financial penalty—they must accurately identify who requires it. Achieving this through the NCSR database is ideal. With the integration of the NCSR Healthcare Provider Portal into private practice management software [2, 7], access in private practice is relatively easy [8]. However, the situation is more difficult in public antenatal care with its large and frequently changing staff. For security reasons, NCSR practitioner access is restricted to those with a Medicare Provider Number (mostly doctors, a few nurse practitioners) [8] or staff registered to act on their behalf. Identification requirements must be met and an account created. Hospital‐based midwives are not permitted their own access, while junior doctors (residents, trainees) on brief rotations mostly have not sought access. Even for clinicians possessing NCSR access, logging in and locating the woman's record without short‐cut software, adds critical minutes in a busy clinic. Needing to confirm CST eligibility via the NCSR therefore represents a barrier to offering CST screening in Australian public antenatal care.

An alternative is to use the woman's self‐reported last CST—fast, easy and a routinely asked pregnancy booking‐in question. However, studies in non‐pregnant women suggest the self‐reported last CST may sometimes be unreliable. In particular, patients not uncommonly over‐report being CST‐current when, in fact, they are overdue [9, 10, 11, 12, 13, 14, 15]. No data exist on the reliability of self‐reported last CST in pregnant women. The implication is to evaluate whether clinicians can simply trust women's recall of cervical screening or if the NCSR needs to be checked for everyone.

The aim of our study was to compare a pregnant woman's self‐reported year of last CST with the date recorded in the NCSR. The question we wanted to answer was not whether there was an exact year match between the databases, but rather, were they concordant for the clinical question of whether a CST was due and should be offered at the early pregnancy booking in visit or not. We also wanted to explore if concordance (self‐report reliability) varied with sociodemographic factors.

## 2 Materials and Methods

### 2.1 Study Design, Study Population and Data Sources

A retrospective cross‐sectional study was performed in an Australian tertiary hospital from 1 June to 30 November 2023. We chose this period as it was almost 6 years after the transition to a 5‐year screening cycle. In addition, prior to February 2024, our hospital essentially never offered CST to pregnant women and never checked the NCSR database, meaning the self‐reported last CST in 2023 was uncontaminated by verification. Regarding study size, no data exist for pregnant women. Of the 22 studies in a recent meta‐analysis involving non‐pregnant women, the median study size was 463 (IQR 251–599) and only two studies exceeded 1000 patients. We felt that a 6‐month sample (1500–2000 women) should be both informative and feasible.

The woman's exact date of last CST, as accessed from the NCSR, served as the reference. The woman's self‐reported year of last CST was sourced from the local eMaternity database. This database is used by many hospitals in New South Wales, with extensive data prospectively entered at pregnancy booking in and at birth. All women who booked in for pregnancy over the 6 months were included if they were ≥ 25 years old, Medicare‐eligible and public patients receiving antenatal care at the hospital. Participants were excluded if < 25 years, not Medicare‐eligible, receiving antenatal care privately, had no self‐reported CST date in the eMaternity database or no record in the NCSR database.

Patient socio‐demographic data was obtained from eMaternity. Australian Bureau of Statistics classifications were used for country of birth groupings [1] and postal‐code derived socioeconomic quintile [16]. Mental health conditions and substance use were self‐reported.

### 2.2 Accuracy Measures

Being CST‐current on the day of pregnancy booking‐in was defined as the NCSR record showing a normal CST within 5 years or being current with recommended follow‐up after an abnormal result. CST‐overdue was > 5 years since last CST or overdue for any recommended follow‐up. CST‐never‐screened was no evidence of screening in the woman's NCSR record.

Concordance between the self‐reported and NCSR last CST date was interpreted, not in terms of an exact year match, but in terms of the answer to the clinical question of whether a CST was due and should be offered at the booking‐in visit or not. By established convention [14], specificity was the ability of self‐report to identify that a CST was needed, while sensitivity was the ability of self‐report to identify that CST screening was current. An over‐report was a false‐positive for CST‐currency, that is, self‐reporting as CST‐current when the NCSR reported CST‐overdue/never‐screened. An under‐report was the opposite. Positive predictive value (PPV) was the proportion of self‐reports of being CST‐current that were validated by the NCSR, while negative predictive value (NPV) was the proportion of self‐reports of being not current that were validated.

### 2.3 Statistical Analysis

Statistical analysis was performed in SPSS 28.0 (IBM). Statistical methods included logistic regression (categorical variables) and Mann–Whitney *U* test (continuous and ordinal variables) for univariate analysis. In undertaking multivariable analysis, a multivariable logistic regression model using a stepwise backwards elimination protocol was used, with variables maintaining a *p*‐value < 0.15 kept in the model. In uni‐ and multivariable analysis, a *p*‐value < 0.05 was considered significant.

### 2.4 Ethics

The study was approved by the Western Sydney Local Health District Research/Education Network Human Research Ethics Committee (2404‐08 QA).

## 3 Results

**FIGURE 1:**
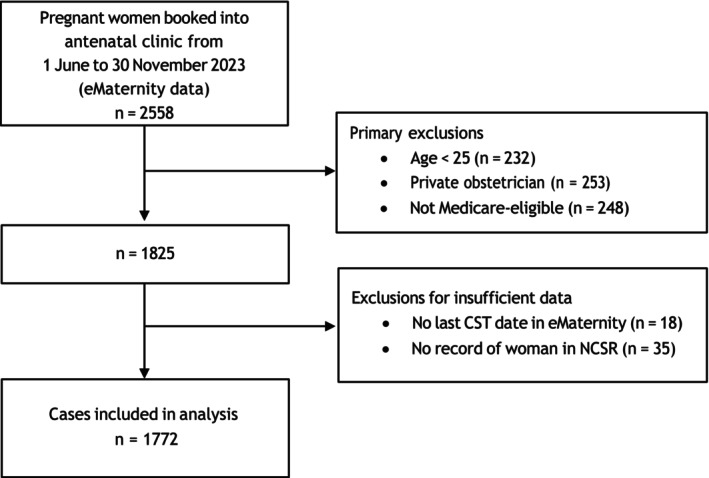
Flowchart of participant inclusion. CST, cervical screening test; NCSR, National Cancer Screening Register.

Over the study period, 2558 women booked into hospital for pregnancy care. After excluding those under 25 years (*n* = 232), Medicare‐ineligible (*n* = 248) or receiving antenatal care privately (*n* = 253), 1825 remained. With further exclusions for missing eMaternity (*n* = 18) or NCSR (*n* = 35) data, the final cohort was 1772 (Figure 1).

The median age was 33 years (IQR 29–36). About 60% (*n* = 1072) were overseas‐born, while 0.7% (*n* = 12) identified as First Nations people. Overall, 910 (51%) were CST‐current, 420 (24%) were overdue and 442 (25%) were never‐screened. Comparing 1072 overseas‐born women with 700 Australian‐born women, the respective figures were CST‐current 47% versus 57%, CST‐overdue 21% versus 28% and CST‐never 32% versus 15% (Table S1). Of CST‐overdue women, almost three‐quarters (*n* = 303; 72%) were more than 12 months overdue.

In terms of recalling the exact year of last CST, of the total 1772 women, 1031 (58%) of self‐reports matched the NCSR record. In terms of needing or not needing a CST, 1420 (80%) demonstrated concordance with the NCSR, comprising 802 (45%) who were CST‐current and 618 (35%) who were not current. Over‐reporting CST currency occurred in 244 (14%) and under‐reporting in 108 (6%).

**FIGURE 2:**
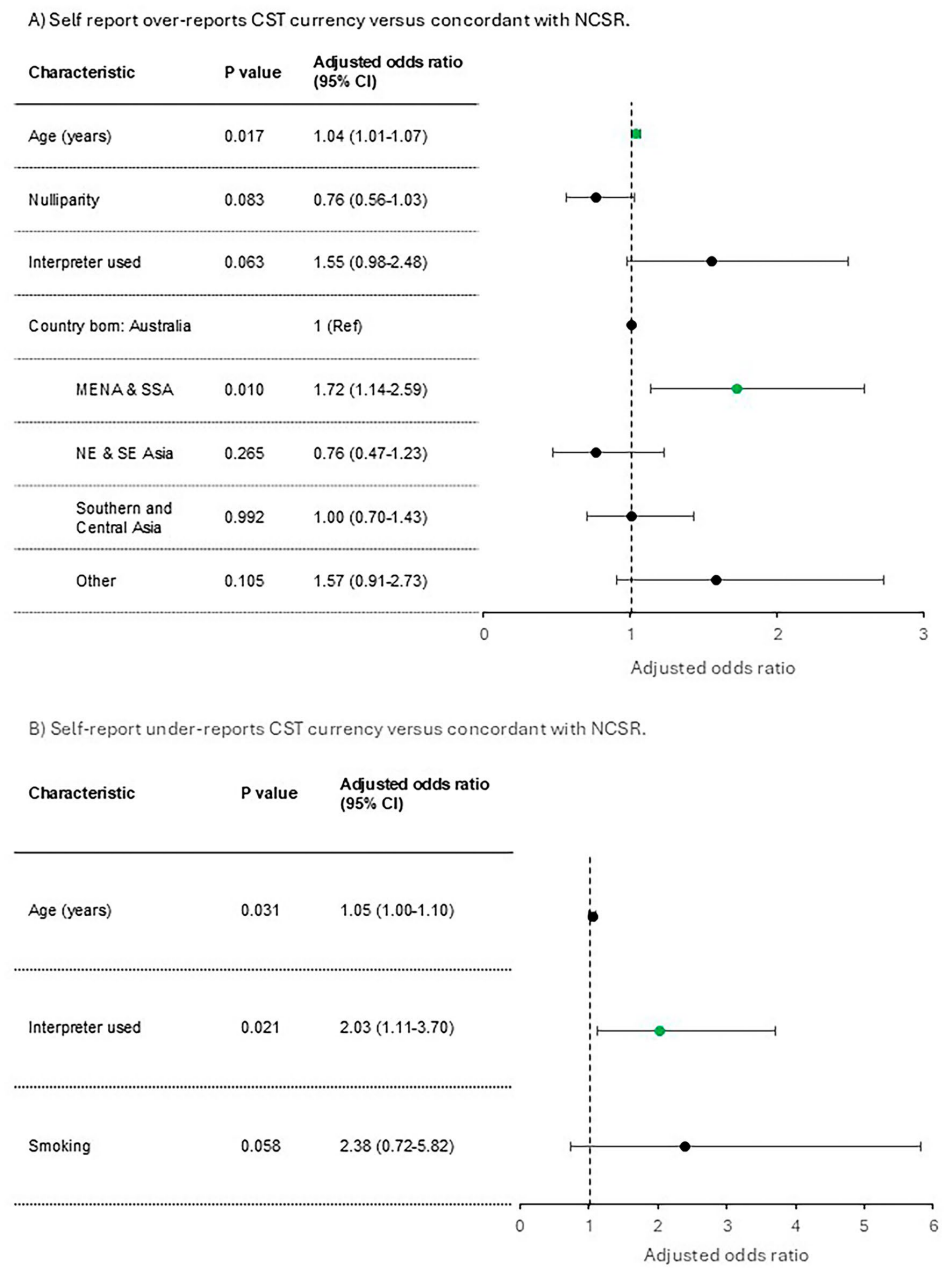
Multivariable analysis of associations with over‐ and under‐reporting CST currency versus being concordant with the NCSR record. Forest plot based on multivariable analysis of variables associated with differences among women who (A) over‐report cervical screening currency compared with concordant self‐report and (B) under‐report cervical screening currency compared with concordant self‐report. The multivariable analysis was limited to variables that had a *p*‐value < 0.15 in the univariable analysis (a stepwise backwards elimination protocol was used for the multivariable logistic regression model). CI, confidence interval; MENA, Middle East & North Africa; NE, north‐east; SE, south‐east; SSA, Sub‐Saharan Africa.

In unadjusted analysis (Table 1), over‐reporting was associated with slightly older age, being born in the Middle East/Africa (over‐report rate 21%), and use of an interpreter. Over‐reporting rates for South Asian (13%) and North‐East/South‐East Asian women (11%) were similar to Australian‐born women (12%). Nulliparity was associated with a lower likelihood of over‐reporting. After multivariable analysis (Figure 2), over‐reporting was associated with older age (adjusted odds ratio [aOR] 1.04, 95% confidence interval [CI] 1.01–1.07) and being born in the Middle East/Africa (aOR 1.72 [95% CI 1.14–2.59]). There was a trend to association with interpreter‐use (aOR 1.55 [95% CI 0.98–2.48]). Under‐reporting was more common with older age (aOR 1.05 [95% CI 1.00–1.10]) and interpreter use (aOR 2.03 [95% CI 1.11–3.70]). There were otherwise no socio‐demographic differences between the groups.

**TABLE 1:** Demographic characteristics and unadjusted odds of over‐ and under‐reporting CST currency versus being concordant with the NCSR record.

Characteristic	Self‐report is concordant with NCSR (*n* = 1420, 80%)	Self‐report over‐reports being CST‐current (*n* = 244, 14%)	Self‐report under‐reports being CST‐current (*n* = 108, 6%)	Over‐report versus concordant		Under‐report versus concordant	
				*p*	OR (95% CI)	*p*	OR (95% CI)
Age (median, IQR)	32 (29–36)	33 (30–37)	33 (30–37)	**0.003**		**0.029**	
Nulliparous	541 (38.1)	70 (28.7)	31 (28.7)	**0.005**	0.65 (0.49–0.74)	0.053	0.65 (0.43–1.01)
Overseas‐born	842 (59.6)	159 (65.6)	64 (60.2)	0.081	1.29 (0.97–1.71)	0.91	1.02 (0.69–1.53)
Country of birth^a^							
Australia	578 (40.7)	85 (34.8)	44 (40.7)	1 (ref)		1 (ref)	
Southern & Central Asia	405 (28.5)	66 (27.0)	29 (26.9)	0.543	1.11 (0.79–1.57)	0.805	0.94 (0.58–1.53)
NE & SE Asia	196 (13.8)	27 (11.1)	20 (18.5)	0.704	0.92 (0.58–1.45)	0.299	1.34 (0.77–2.33)
MENA & SSA	162 (11.4)	47 (19.3)	10 (9.3)	**0.001**	2.00 (1.35–2.97)	0.562	0.81 (0.40–1.65)
Other	79 (5.6)	19 (7.8)	5 (4.6)	0.071	1.65 (0.96–2.86)	0.705	0.83 (0.32–2.16)
Interpreter used	98 (7)	28 (11.5)	14 (13)	**0.014**	1.75 (1.12–2.73)	**0.022**	2.01 (1.11–3.65)
Two lowest socioeconomic quintiles^b^	418 (29.4)	81 (33.2)	35 (32.4)	0.24	1.19 (0.89–1.59)	0.52	1.15 (0.76–1.75)
Mental health condition^c^	289 (20.4)	44 (18)	26 (24.1)	0.40	0.86 (0.61–1.22)	0.36	1.24 (0.78–1.97)
Substance use in pregnancy^c^							
Smoking	35 (2.5)	7 (2.9)	6 (5.6)	0.71	1.17 (0.51–2.66)	0.063	2.33 (0.96–5.66)
Alcohol	11 (0.8)	1 (0.4)	2 (2)	0.54	0.53 (0.07–4.10)	0.26	2.42 (0.53–11.05)
Illicit drugs	9 (0.6)	1 (0.4)	2 (2)	0.68	0.65 (0.08–5.12)	0.17	2.96 (0.63–13.87)

*Note:* Except where otherwise specified, results represent *n* (%). A *p* value < 0.05 is considered significant (in bold).

Abbreviations: CST, cervical screening test; MENA, Middle East & North Africa; NCSR, national cancer screening register; NE, north‐east; Ref, reference; SE, south‐east; SSA, Sub‐Saharan Africa.

^a^ Country of Birth is classified according to Australian Standard Classification of Cultural and Ethnic Groups (ASCCEG), 2019, Australian Bureau of Statistics (abs.gov.au). ‘Other’ encompasses Europe, the Americas, Oceania and Unknown. Over‐report rates by country of birth calculable from this table: Australia 12% (85/707), Southern & Central Asia 13% (66/500), SE & NE Asia 11% (27/243), MENA/SSA 21% (47/219), Other 18% (19/103); Overall 14% (244/1772).

^b^ Index of Relative Socioeconomic Disadvantage which is based on postal code. *Source:* Socioeconomic Indexes for Areas (SEIFA) reference period 2021, released 2023. Australian Bureau of Statistics (abs.gov.au).

^c^ Self‐reported.

Among the 862 women who were CST‐overdue/never‐screened, 618 accurately reported needing a CST (specificity 72%) while 244 (28%) erroneously over‐reported being CST‐current. Of the 910 women who were CST‐current, 802 accurately reported this (sensitivity 88%) while 108 (12%) erroneously under‐reported their CST status. Overall, 77% of self‐reports for being CST‐current were validated by the NCSR (PPV) and 85% of self‐reports for being CST‐overdue/never‐screened were validated (NPV) (Table 2).

**TABLE 2:** Accuracy of patient self‐report for being CST‐current compared to the NCSR record.

	NCSR:CST‐current		NCSR:CST‐overdue/never‐screened		Total
	*n*	%	*n*	%	
Self‐report: CST‐current	802	88%^a^	244	28%^b^	1046
Self‐report: CST‐overdue/never‐screened	108	12%^c^	618	72%^d^	726
TOTAL	910	51%	862	49%	1772
Sensitivity^a^	88%	95% CI: 86%–90%			
Specificity^d^	72%	95% CI: 69%–75%			
Positive predictive value (PPV)^e^	77%	95% CI: 75%–79%			
Negative predictive value (NPV)^f^	85%	95% CI: 83%–87%			

Abbreviations: CI, confidence interval; CST, cervical screening test; NCSR, national cancer screening register.

^a^ Sensitivity: Ability of self‐report to identify that CST was not needed.

^b^ Over‐report for CST‐currency: CST needed (CST‐overdue/never) but self‐reported as CST‐current (100% minus specificity).

^c^ Under‐report for CST‐currency: CST not needed (CST‐current) but self‐reported as CST‐overdue/never.

^d^ Specificity: Ability of self‐report to identify that CST was needed.

^e^ PPV: Proportion of self‐reports for being CST‐current that were validated by the NCSR.

^f^ NPV: Proportion of self‐reports for being CST‐overdue/never that were validated by the NCSR.

## 4 Discussion

### 4.1 Overall Findings

To exploit the opportunity pregnancy offers for CST catch‐up in public antenatal care while simultaneously avoiding the financial penalty incurred by screening too soon, clinicians must accurately identify those requiring a CST. In this Australian, multiethnic, Medicare‐eligible population, concordance between the NCSR record and a woman's self‐report with respect to needing or not needing a CST was 80%. However, about 1 in 7 (14%) erroneously over‐reported and 1 in 16 (6%) under‐reported being CST‐current. Over‐reporting CST currency is the greater clinical concern. While corresponding to 14% of the entire cohort, over‐reporting represented 28% of those women (half the total cohort) needing a CST (specificity 72%). Already at increased risk of cervical cancer, these women would then miss the opportunity to catch up.

### 4.2 Comparison With Other Studies

Since 1976 [17] and across a range of countries and populations, cervical screening self‐report studies have consistently demonstrated high rates of over‐reporting [9, 10, 11, 12, 13, 15, 18, 19, 20]. A recent meta‐analysis of 22 studies/61,000 women revealed an average over‐reporting rate of 52% (range 19%–82%) (specificity of 48%). All included studies examined the Pap test and none specifically examined for HPV screening [14], while self‐collect CST is far too new for self‐report reliability data. Our over‐reporting rate of 28% is at the lower end of the meta‐analysis range. This may reflect our focus on CST‐currency rather than exact‐year accuracy, a 5‐year screening interval improving the likelihood a previously‐screened patient was still CST‐current [9, 14] and a relatively large CST‐never‐screened group—assuming ‘never’ is easier to recall than a date.

### 4.3 Over‐Reporting in Other Cancer Screening

Over‐reporting is also common with breast and bowel cancer screening, but at lower rates than cervical cancer screening [10, 11, 12, 13, 14, 15]. Researchers speculate that, compared to a mammogram or faecal occult blood test (or colonoscopy), which are distinct stand‐alone investigations, a woman undergoing a CST might not easily distinguish the test from a pelvic, speculum or swab examination for another reason.

### 4.4 Socio‐Demographic Associations With Over‐Reporting CST‐Currency

In our study, the likelihood of over‐report was reasonably distributed across the population. After adjustment in multivariable analysis, Middle Eastern/African women had increased over‐report risk, while interpreter‐use showed a trend to over‐report (both aOR < 2). These small groups overlap and Middle Eastern/African women at 21% over‐report rate represented many fewer patients than Australian‐born women at 12% over‐report. Regarding the minor over‐report increase with slightly older age (aOR 1.04), we do not believe it is useful for public health or clinical decision‐making. No other socio‐demographic factors were associated with over‐report. The CST‐validation literature is heterogeneous with respect to population studied and also question explored (CST ever vs. never, CST time interval/currency, CST ever abnormal). Associations with CST over‐reporting are inconsistent. While some described a higher likelihood of over‐reporting with smoking [12], immigrant or ethnic minority [10] or lower socioeconomic status [13], others [9, 15], including the recent meta‐analysis [14], reported no associations.

### 4.5 Strengths and Limitations

Regarding strengths, to our knowledge, ours is the first CST self‐report validation study in pregnant women. The gold standard reference was the NCSR, a well‐resourced, regularly‐updated national database. The study examined data from all women booking‐in for public pregnancy care over 6 months rather than ad‐hoc responders to a survey request, resulting in low selection bias. The cohort was multi‐ethnic, mixed‐income and of sufficient size to demonstrate that erroneously over‐reporting being CST‐current is a problem across all socio‐demographic groups. While fewer of our women were CST‐current than the national average, decades of publications confirm that our findings on over‐reporting are broadly generalisable.

The study has limitations. First, by its nature it is retrospective. However, the eMaternity booking‐in data was both recent and prospectively recorded, while NCSR data is updated frequently. Second, while the numbers were reasonably large, study size was limited by the time required to check each woman's NCSR record. Third, while comparison of reliable databases offers considerable benefits, it does not permit exploration of the reasons for CST over‐reporting in the way a survey study might. Fourth, the numbers of First Nations women were too small for analysis. Finally, we have no information about the situation with Medicare‐ineligible women.

### 4.6 Future Directions: Improving CST Awareness and Self‐Reporting Accuracy

While some groups (socioeconomic disadvantage, limited English, identifying as First Nations or LGBTQI+) may need special focus to ensure equity [21], improving CST awareness and self‐report accuracy requires targeting all screen‐eligible women. First, a woman must comprehend that she has undergone cervical screening versus a different gynaecological assessment. Self‐collect CST should make screening more memorable, but it must be enhanced by good clinician communication and written information. Second, women should be sent their CST results. Australian women participating in breast [22] and bowel [23] cancer screening receive their results by post after 2 weeks. Those having ultrasound at private practices often obtain indefinite access to their images and reports via an App [24]. In the United Kingdom, cervical screening results are sent to the woman [25]. However, in Australia, cervical screening results are currently sent only to the referring practitioner [26]. Third, women should be as easily able to access their CST information online as their healthcare practitioner.

Steps are being taken to improve participant access to the NCSR. The NCSR provides a phone number and a Participant Portal [27] where women can locate the date of their last CST (but not the result) and when the next CST is due. However, portal access requires first providing the detailed information required to set up a MyGov account and then separately creating an NCSR link by providing further identification. While privacy and security demand such measures, gaining access takes time and good written English skills. Additionally, it may not always be easily accomplished on a mobile phone. The anecdotal impression that few screening participants currently access this portal is confirmed by usage data. As of August 2024, the portal had 92 000 combined cervical‐ and bowel‐screen registered users, representing 1.3% of the combined 4.7 million cervical‐ and 2.4 million bowel‐screen participants in the most recently‐reported screening cycles [7].

Our findings confirm the almost 50 years of prior studies acrosssss a range of populations that self‐reported CST‐currency can be unreliable and that over‐reporting CST currency risks missing exactly the women most in need of screening. System improvements that empower Australian women can reverse this. These include providing adequate CST explanation (verbal and written) at the time of collection, routinely sending women their CST results and further progressing the pioneering work of the NCSR to ensure the Participant Portal is both well‐publicised and easy to access. These measures will ensure patient self‐reported last CST date is reliable and thus remove a barrier to cervical screening in Australian public antenatal care.

## Conflicts of Interest

The authors declare no conflicts of interest.

## Supporting information


**Table S1:** Demographic characteristics and unadjusted odds of being CST‐overdue or CST‐never versus being CST‐current based on the NCSR record.
