# Supplementary material for: Accuracy of Self‐Reported Cervical Screening Status Among Pregnant Women
Source: Aust N Z J Obstet Gynaecol. 2025 Nov 26;66(1):e70057. doi: 10.1111/ajo.70057 (PMC12873515; doi:10.1111/ajo.70057)
Supplement: Supplementary file 1 — Table S1: Demographic characteristics and unadjusted odds of being CST‐overdue or CST‐never versus being CST‐current based on the NCSR record. [file AJO-66-0-s001.docx]

**Supplementary Table 1: Demographic characteristics and unadjusted odds of being CST-overdue or CST-never versus being CST-current based on the NCSR record.**

|  | | **Total**  **n=1772** | **CST-Current**  **n=910, (51%)** | **CST -Overdue**  **n=420**  **(24%)** | **CST-Never-screened**  **n=442**  **(25%)** | **CST-Overdue/Never-screened**  **Versus CST-Current** | | **CST-Overdue versus**  **CST Current** | | **CST Never-screened**  **versus**  **CST Current** | |
| --- | --- | --- | --- | --- | --- | --- | --- | --- | --- | --- | --- |
|  |  |  |  |  |  | P value | OR (95% CI) | P value | OR (95% CI) | P value | OR (95% CI) |
| Age^†^ | | 33 (29-36) | 33 (30-36) | 33 (30-37) | 31 (28-34) | **<0.001** |  | 0.43 |  | **<0.001** |  |
| Nulliparous | | 642 (36) | 301 (33) | 137 (33) | 204 (46) | **0.005** | 1.32 (1.09-1.61) | 0.86 | 0.98 (0.77-1.25) | **<0.001** | 1.73 (1.37-2.19) |
| Overseas-born | | 1072 (60) | 509 (56) | 223 (53) | 340 (77) | **<0.001** | 1.48  (1.22-1.80) | 0.33 | 0.89  (0.71-1.13) | **<0.001** | 2.63  (2.03-3.40) |
| Interpreter Used | | 140 (8) | 61 (7) | 21 (5) | 58 (13) | 0.055 | 1.40  (0.99-1.99) | 0.23 | 0.73  (0.44-1.22) | **<0.001** | 2.10  (1.44-3.07) |
| Two Lowest Socio-economic Quintiles^‡^ | | 533 (30) | 242 (26) | 126 (30) | 165 (37) | **<0.001** | 1.41 (1.15-1.73) | 0.168 | 1.20 (0.93-1.54) | **<0.001** | 1.64 (1.29-2.10) |
| Mental Health Condition^§^ | | 359 (20) | 227 (25) | 81 (19) | 51 (12) | **<0.001** | 0.54  (0.43-0.69) | **0.023** | 0.72  (0.54-0.96) | **<0.001** | 0.39  (0.28-0.55) |
| Substance Use in Pregnancy^§^ | |  |  |  |  |  |  |  |  |  |  |
| Smoking | 48 (3) | 23 (3) | 16 (4) | 9 (2) | 0.63 | 1.15  (0.65-2.05) | 0.20 | 1.53  (0.80-2.92) | 0.58 | 0.80  (0.37-1.75) |  |
| Illicit drugs | 12 (0.7) | 8 (0.9) | 3 (0.7) | 1 (0.2) | 0.29 | 0.53  (0.16-1.75) | 0.76 | 0.81  (0.21-3.07) | 0.17 | 0.26  (0.03-2.05) |  |
| Alcohol | 14 (0.8) | 8 (0.9) | 3 (0.7) | 3 (0.7) | 0.66 | 0.79  (0.27-2.29) | 0.76 | 0.81  (0.21-3.07) | 0.70 | 0.77  (0.20-2.92) |  |

Except where otherwise specified, results represent n (%). A *p* value < 0.05 is considered significant (in bold).

^†^Median, IQR

^‡^Index of Relative Socioeconomic Disadvantage which is based on postal code. Source: Socioeconomic Indexes for Areas (SEIFA) reference period 2021, released 2023. Australian Bureau of Statistics ([abs.gov.au](https://www.abs.gov.au/statistics/people/people-and-communities/socio-economic-indexes-areas-seifa-australia/latest-release#index-of-relative-socio-economic-disadvantage-irsd-)).

^§^ Self-reported

Additional information calculable from the table: rates of CST-current, CST-overdue and CST-never in 1072 overseas-born women were 47%, 21% and 32% respectively, while in 700 Australian-born women the rates were 57%, 28% and 15%.

CI – confidence interval; CST – cervical screening test; NCSR – national cancer screening register; OR – odds ratio.
